# Sulfated Polysaccharides from *Chaetoceros muelleri*: Macromolecular Characteristics and Bioactive Properties

**DOI:** 10.3390/biology11101476

**Published:** 2022-10-08

**Authors:** Valeria Miranda-Arizmendi, Diana Fimbres-Olivarria, Anselmo Miranda-Baeza, Karla Martínez-Robinson, Agustín Rascón-Chu, Yubia De Anda-Flores, Jaime Lizardi-Mendoza, Mayra A. Mendez-Encinas, Francisco Brown-Bojorquez, Rafael Canett-Romero, Elizabeth Carvajal-Millan

**Affiliations:** 1 Research Center for Food and Development (CIAD, AC), Carretera Gustavo Enrique Astiazarán Rosas No. 46, Col. La Victoria, Hermosillo 83304, Sonora, Mexico; 2 Department of Scientific and Technological Investigations (DICTUS), University of Sonora, Blvd. Luis Donaldo Colosio, S/N, Hermosillo 83000, Sonora, Mexico; 3 Laboratory of Cultivation Technologies of Marine Organisms, State University of Sonora, Blvd. Manlio Fabio Beltrones No. 810, Col. Bugambilias, Navojoa 85875, Sonora, Mexico; 4 Department of Chemical Biological and Agropecuary Sciences (DCQBA), University of Sonora, Av. Universidad e Irigoyen, S/N, Caborca 83621, Sonora, Mexico; 5 Department of Polymers and Materials (DIPM), University of Sonora, Rosales and Blvd. Luis D. Colosio, Hermosillo 83000, Sonora, Mexico; 6 Department of Research and Postgraduate in Food (DIPA), University of Sonora, Rosales and Blvd. Luis D. Colosio, Hermosillo 83000, Sonora, Mexico

**Keywords:** microalgae, sulfated polysaccharides, molecular conformation, microstructure, bioactivity

## Abstract

**Simple Summary:**

Algae are an important source of bioactive compounds. The interest in microalgae is increasing due to their high-value products and the advantage of biomass cultivation under controlled conditions. Polysaccharides are released by algae and microalgae species and have been reported to have bioactivities found beneficial to human health. Despite the recognized importance of these organisms, the structure of polysaccharides in microalgae has been practically unexplored in contrast to that of macroalgae. Only a few microalgae polysaccharide structures have been solved due to the difficulties in the extraction of pure samples and the complexity of their chemical structures. Reports emphasize how the molecular weight, the content of sulfate groups, and the negative charge may be responsible for their multiple bioactivities. To better understand the uses and potential applications of extracellular polysaccharides, it is necessary to know their structure and physicochemical properties, which include molecular weight and chain conformation, since they are decisive in their biochemical behavior.

**Abstract:**

In the present study, a culture of *Chaetoceros muelleri*, a cosmopolitan planktonic diatom microalga present in the Sea of Cortez, was established under controlled laboratory conditions. A sulfated polysaccharide (CMSP) extraction was carried out from the biomass obtained, resulting in a yield of 2.2% (w/w of dry biomass). The CMSP sample was analyzed by Fourier transform infrared spectroscopy, showing bands ranging from 3405 to 590 cm^−1^ and a sulfate substitution degree of 0.10. Scanning electron microscopy with elemental analysis revealed that the CMSP particles are irregularly shaped with non-acute angles and contain sulfur. High-performance liquid chromatography coupled to a dynamic light-scattering detector yielded molecular weight (*Mw*), polydispersity index (*PDI*), intrinsic viscosity [*η*], and hydrodynamic radius (*Rh*) values of 4.13 kDa, 2.0, 4.68 mL/g, and 1.3 nm, respectively, for the CMSP. This polysaccharide did not present cytotoxicity in CCD-841 colon cells. The antioxidant activity and the glycemic index of the CMSP were 23% and 49, respectively, which gives this molecule an added value by keeping low glycemic levels and exerting antioxidant activity simultaneously.

## 1. Introduction

The variety of organisms in the marine environment represents a vast source of valuable resources. Among them, algae are an important source of bioactive compounds, such as polysaccharides, proteins, peptides, lipids, amino acids, and mineral salts [1]. Polysaccharides and sulfated polysaccharides are frequent components of the cell walls of algae. These biomacromolecules are often closely related to pharmacological activities as anticoagulant, antioxidant, antitumor, and immunomodulatory agents [2,3]. Therefore, in recent decades, sulfated polysaccharides of algal origin have gained much interest as functional additives in the pharmaceutical, food, and cosmetic industries. However, research with algae has been mainly limited to analyzing the bioactivity of sulfated polysaccharides, while studies on the structure–function relationship of these polysaccharides are relatively scarce [4].

Reported studies emphasize how molecular weight, content of sulfate groups, and negative charge may be responsible for the multiple bioactivities of these polysaccharides. Sulfated polysaccharides synthesized by several macroalgae are heterogeneous and structurally different, making their research challenging. Despite the recognized importance of these organisms, the structure of the polysaccharides in microalgae has been almost completely unexplored in contrast to that of macroalgae. Only a few microalgae polysaccharide structures have been identified due to the difficulties in extracting pure samples and the complexity of their chemical structures [5]. However, unlike macroalgae, microalgae have the advantage of being cultivated under controlled conditions. The controlled culture of microalgae could allow their polysaccharides’ chemical composition, structure, and rheological behavior to be more homogeneous, regardless of the harvesting periods [6].

*Chaetoceros muelleri* is a cosmopolitan planktonic diatom microalga found in the Gulf of California, Mexico. Due to its rapid growth and easy maintenance, it has been used in aquaculture to feed shrimp larvae in commercial hatcheries in many regions [7]. The biomass of *C. muelleri* can generate high-value products, such as long-chain polyunsaturated fatty acids, proteins, and carbohydrates, making it one of the most important species of microalgae in the aquaculture industry. Several studies have been reported on the production of fatty acids and reserve carbohydrates in this species [8]. However, to date, there is no information on its sulfated polysaccharides.

In order to have a better understanding of the different potential applications of polysaccharides, it is necessary to consider properties such as their macromolecular characteristics, microstructure, and bioactivity since these determine their functionality [9]. Therefore, studying the structure–function relationship of polysaccharides from new sources, such as those from some microalgae, may represent the starting point for developing new bioactive products or biomaterials. Thus, this research aims to generate new insights into the macromolecular characteristics and bioactive properties of sulfated polysaccharides from the diatom *C. muelleri*.

## 2. Materials and Methods

### 2.1. Microalga Cultivation and Polysaccharide Extraction

The microalga *C. muelleri* was obtained from the strain collection of the Department of Scientific and Technological Research (DICTUS) of the University of Sonora. A 200 L culture was carried out under controlled conditions in the Laboratory of Technologies for the Cultivation of Marine Organisms of the State University of Sonora using the “F” medium of Ryther and Guillard [10], white light illumination (3500 lux), 23 °C temperature, and continuous aeration. Daily cell counts were performed in triplicate using a Bright-line Neubauer hemacytometer with a 0.1-mm deep chamber, as previously described [8]. The biomass was flocculated with Al_2_(SO_4_)_3_ to facilitate the precipitation of the cells and harvested at the beginning of the stationary phase of the culture, established as the third day after inoculum (which was assigned as day 0). The recovered biomass was lyophilized. Subsequently, 20 g of lyophilized biomass was resuspended in 200 mL of distilled water, and the mixture was stirred for 1 h at room temperature. The suspended biomass was centrifuged for 10 min at 20,000× *g* to obtain a cell pellet. A volume of 200 mL of 95% ethanol was added to the resultant cell pellet and then heated to 42 °C and shaken for 1 h at 350 rpm to remove pigments and lipids. After the centrifugation, the cell pellet was washed three times with 100 mL of 95% ethanol to remove pigments and proteins. The cell pellet was suspended in 400 mL of sulfuric acid (50 mM) in a water bath at 60 °C for 30 min. The supernatants were collected by centrifugation and precipitated with four volumes of 95% ethanol at −20 °C for 24 h. The precipitate was recovered by centrifugation and washed by solvent exchange (ethanol–acetone) to eliminate any Al_2_(SO_4_)_3_ remnants, subsequently resuspended again, filtered (45 µm), and lyophilized to obtain the *C. muelleri* sulfated polysaccharides (CMSPs) [11].

### 2.2. Chemical Analysis

The protein content was determined using the Bradford method [12]. The carbohydrates were quantified by the phenol–sulfuric acid test [13]. An ash analysis was performed according to the AOAC 920,153 method [14].

### 2.3. Attenuated Total Reflectance-FourierTransform Infrared (ATR-FTIR) Spectroscopy

An ATR-FTIR analysis of the CMSP dry powder was performed on a Nicolet iS50 FTIR spectrometer (Madison, WI, USA). The spectrum was registered from 4000 to 400 cm^−1^ [15]. The degree of sulfation was estimated from the ATR-FTIR spectrum [16].

### 2.4. Microstructure and Elemental Composition

The microstructure of the CMSP was studied by scanning electron microscopy (SEM) (JEOL 5410LV, JEOL, Peabody, MA, USA) at low voltage (20 kV). A SEM image was obtained in the secondary electron imaging (SEI) mode. The chemical composition was performed using energy-dispersive X-ray spectroscopy (EDX) (INCA-200 Oxford Instruments, Abingdon, Oxfordshire, England).

### 2.5. Macromolecular Characteristics

The weight-average molar mass (*Mw*), the number-average molar mass (*Mn*), the intrinsic viscosity ([*η*]), the hydrodynamic radius (*Rh*), and the polydispersity index (*PDI* = *Mw/Mn*) were determined in the CMSP. A size-exclusion chromatography (SEC) system and a DAWN HELOS-II 8 multi-angle laser-light scattering (MALS) detector coupled with a ViscoStar-II viscometer and a refractive index (RI) Optilab T-rex detector (Wyatt Technology Corp., Santa Barbara, CA, USA) were used. The CMSP was dispersed in 100 mM NaNO_3_/0.02% NaN_3_ at 5 mg/mL and filtered (0.20 µm, Millipore Sigma, Saint Louis, MO, USA). An Agilent HPLC System (G1310B ISO pump, G1329B autosampler, and G1314F variable wavelength detector, Agilent Technologies, Inc., Santa Clara, CA, USA) and the columns Shodex OH-pak SBH-Q-804 and 805 (Shodex Showa Denco K.K., Tokyo, Japan) at a flow rate of 0.7 mL/min (100 mM NaNO_3_/0.02% NaN_3_) at 25 °C were employed. The ASTRA 6.1 software (Wyatt Technology Corp., Santa Barbara, CA, USA) was used.

### 2.6. Cell Line and Culture Conditions

The normal cell line from a human colon, CCD-841 CoN (ATCC^®^ CRL1790TM), was obtained from the American Type Culture Collection (ATCC, Manassas, VA, USA) and grown in a D10F medium, which consisted of Dulbecco’s modified Eagle’s medium (DMEM), supplemented with 10% fetal bovine serum (FBS, Gibco, Life Technologies, Carlsbad, CA, USA), 1% non-essential amino acids, 100 U/mL penicillin, and 100 mg/mL streptomycin. The cells were maintained at 37 °C and 5% CO_2_ in a humidified incubator (Thermo Fischer Scientific, San Jose, CA, USA) [17].

### 2.7. Cytotoxicity

The effect of the CMSP on the proliferation of the human colon cell line, CCD-841 CoN (ATCC^®^ CRL1790™) from the American Type Culture Collection (ATCC, Manassas, VA, USA), was determined following the standard assay of 3-(4,5-dimethylthiazol-2-yl)-2,5-diphenyltetrazolium bromide (MTT) [18,19]. The cells (2 × 10^4^ cells/50 μL) were seeded in 96-well microplates and incubated for 24 h at 37 °C in a 5% CO_2_ atmosphere. The culture medium was used as a control, and the cytotoxic drug 5-fluorouracil (5-FU) was used as a positive control. Aliquots of different concentrations of the CMSP in D10F medium were added to the wells and incubated for 48 h. A total of 10 μL of MTT solution (5 mg/mL) was added to each well in the last 4 h of the incubation period. The cell viability was measured by the resulting colored precipitates formed due to the ability of the metabolically active cells to reduce the tetrazolium salt to form purple crystals. The absorbance of the samples was measured at a test wavelength of 570 nm and a reference wavelength of 650 nm using an ELISA plate reader (Thermo Scientific MultiSkan Go, Madrid, Spain). The results were expressed as the number of viable cells exposed to the treatments compared to the number of control cells (the medium alone) [17].

### 2.8. Antioxidant Activity

To evaluate the antioxidant activity, DPPH (2,2-diphenyl-1-picrylhydrazyl) radical scavenging activity was carried out. Briefly, an ethanolic solution of DPPH (0.1 mM) was prepared, and an aliquot of the sample of 20 mg/mL was added to the DPPH solution (1:1 v/v) [20]. After incubation for 30 min in the dark at room temperature, the absorbance was measured at 517 nm on a Cary 60 UV-Vis spectrophotometer (Agilent Technologies, Santa Clara, CA, USA). The measurements were performed in triplicate, and Vitamin C was used as a positive control. The stabilization of the DPPH radicals by the sulfated polysaccharides was calculated according to the following equation:

DPPH − stabilizing activity (%) = [1 − Asample517nm − Ablank517nm)/Acontrol517nm] × 100

### 2.9. Glycemic Index (GI)

Male Wistar rats (244–278 g), donated by the University of Sonora, were used for the experiment. The organisms were housed individually in stainless steel mesh cages under a controlled environment (23 ± 2 °C, 60 ± 5% relative humidity, and a 12-h day/night cycle). A standard pellet diet (LabDiet 5008) and water *ad libitum* were used during the 15 days of acclimatization. The Research Center for Food and Development (CIAD) Animal Ethical Committee approved the experiment in agreement with the committee’s guidelines for control and supervision of animal experiments (NOM-062-ZOO-1999) in Mexico. After the acclimatization period, the rats were fasted for 10 h and randomly assigned to 2 groups (n = 3/group), the glucose group and the CMSP group, which received 1 mL of glucose or CMSP solution at 25 mg/mL, respectively, by single oral gavage. Blood was taken by tail vein puncture before the treatment and at 15, 30, 45, 60, 90, and 120 min after the administration. Blood glucose levels were measured using an Accu-Chek Active blood glucose meter (Roche, Mannheim, Germany).

The GI value was calculated from the area of the glycemic response curve of the glucose (So) and the CMSP (SCMSP), as reported by Li et al. (2021), as follows:

GI = (SCMSP/So) × 100(1)

The area under the curve was calculated using the OriginPro 2021 (OriginLab Corp., Northampton, MA, USA).

### 2.10. Statistical Analysis

The results are expressed as a mean ± standard deviation (SD) from the triplicates.

## 3. Results

### 3.1. Cell Culture and Polysaccharide Yield

**Figure 1:**
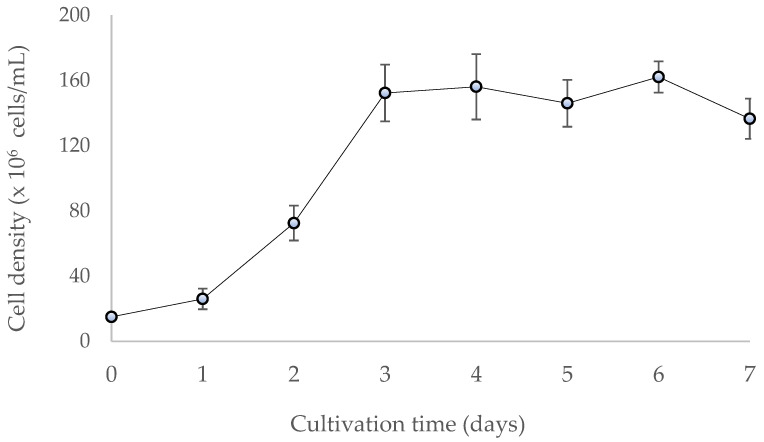
Growth curve of the microalgae *C. muelleri*.

*Chaetoceros* spp. are one of the most important diatoms in the industry, reaching an average concentration of 1 × 10^6^ to 2 × 10^6^ cells/mL and a highly variable biomass production with excellent nutritional value in aquaculture [21]. The resulting cell concentration of the culture in this study reached the stationary phase on day three after inoculation with approximately 1.5 × 10^6^ cells/mL (Figure 1). The biomass yield was 2.8 g/L, and a polysaccharide yield of 2.2% (w/w) was obtained. These values are close to those reported for other diatom species, such as *Gomphonema olivaceum* with 3% (w/w) [22] and *Navicula* sp. with 4% (w/w) [23].

### 3.2. Yield and Chemical Analysis of the CMSP

The extraction yield of the polysaccharides from *C. muelleri* was 2.2% (w/w). The extract obtained was composed of 78% carbohydrates (Table 1). Many environmental factors, including temperature, light exposure, nutrient availability, and salinity, can influence the content of polysaccharides in algae [24]. Residual amounts of protein and ash were also found in the range reported for polysaccharide extractions from other diatoms [5]. In the case of proteins, a value of 5% was found, which coincides with that reported for the diatom *Phaeodactylum tricornutum*, where the protein content determined in the purified samples was around 7% (w/w) [5]. This result suggests a strong association of the cell wall proteins with the polysaccharides in diatoms.

### 3.3. Fourier Transform Infrared Spectroscopy

**Figure 2:**
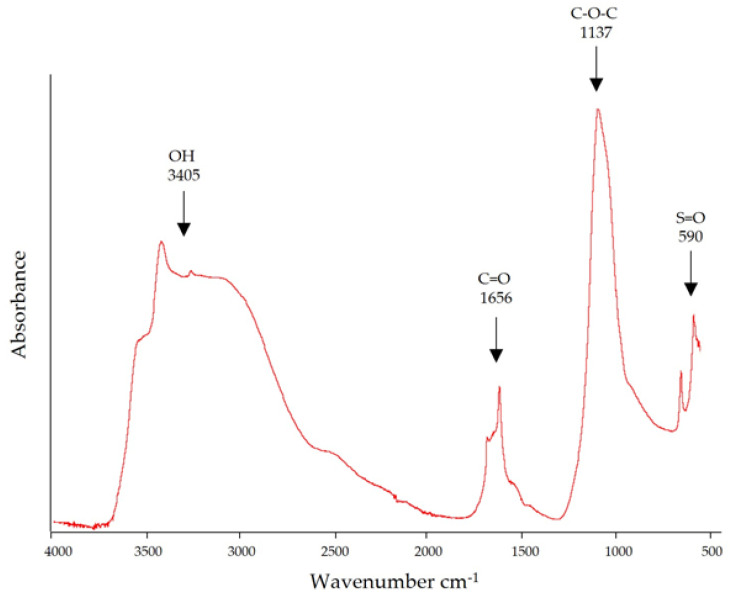
Attenuated Total Reflectance-Fourier Transform Infrared (ATR−TIR) spectrum of the CMSP from *C. muelleri*. The arrows indicate the main absorption bands.

FTIR spectroscopy is an analytical technique for studying molecular structures by identifying vibrations between different atoms present in a sample [25]. Four main bands were identified in the wavenumber range from 3405 to 590 cm^−1^ (Figure 2) in the spectrum of the sulfated polysaccharides from *C. muelleri* analyzed in this study. According to the literature, the 3405 cm^−1^ band is characteristic of the vibration of the OH group and has been reported in polysaccharides extracted from green and brown algae [26,27]. The band detected at 1656 cm^−1^ has been reported in other algae and represents a carboxylic acid group’s carbonyl group (C=O) [28]. The 1035 cm^−1^ band is considered essential in the polysaccharide structure because it corresponds to the stretching vibrations of the glycoside bridge (C–O–C) [29]. Finally, the bands in the range between 622 and 583 cm^−1^ are attributed to the symmetric and asymmetric deformation of the O=S=O bonds of the sulfates in fucoidan extracts [30].

### 3.4. Microstructure and Elemental Composition

**Figure 3:**
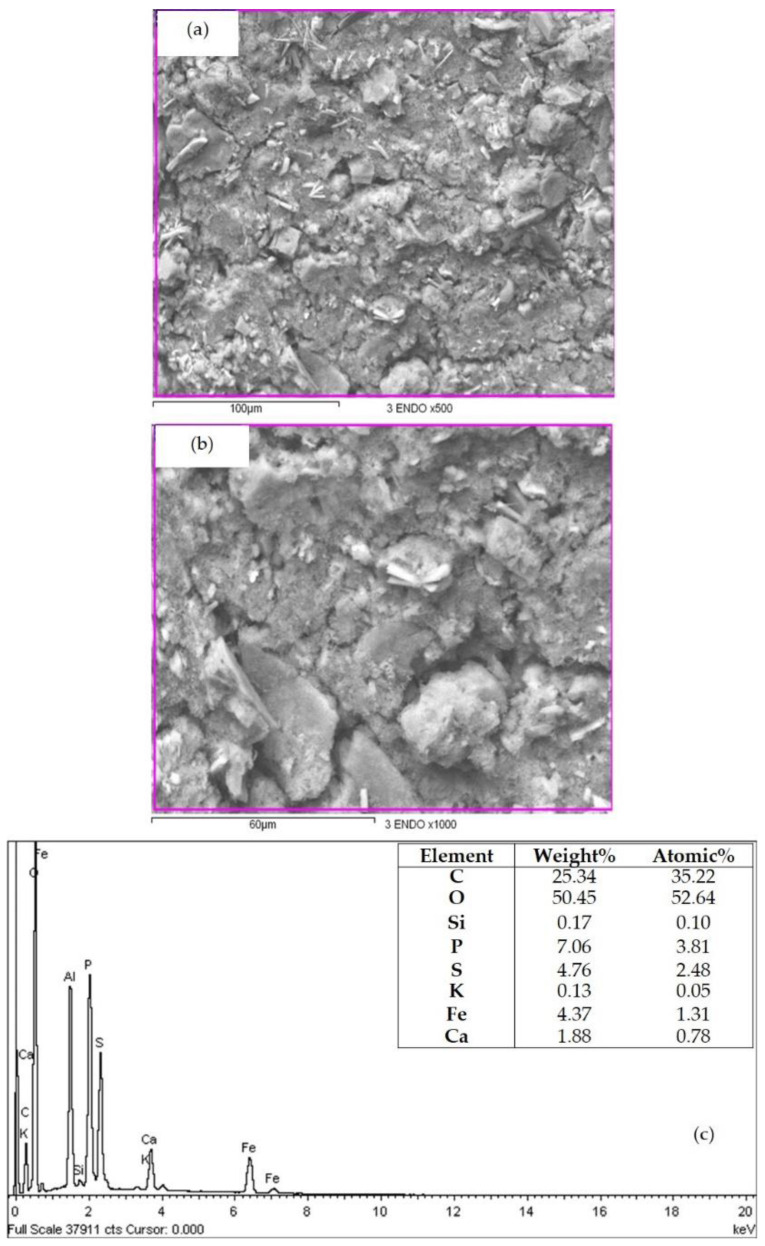
SEM micrographs of the CMSP powder extracted from *Chaetoceros muelleri*. Images are at 500× (**a**) and 1000× (**b**) magnification. Element composition of the CMSP, analyzed by EDX (**c**).

A SEM with elemental analysis (Figure 3a,b) revealed that the dried CMSP particles extracted from *C. muelleri* consist of a uniform powder-like irregularly shaped sample with non-acute angles, which is consistent with the description reported for other sulfated polysaccharides extracted from algae [31]. An element composition analysis showed mainly the presence of carbon, oxygen, sulfur, phosphorus, iron, and calcium (25.34, 50.45, 4.76, 7.06, 4.37, and 1.88%, respectively) (Figure 3c). Elements such as calcium were identified in the sample, probably due to the essential macronutrients and the alkaline earth metal nature of the extraction source. These elements, amongst others, have been described in *Chaetoceros* spp. [32]; however, information of this nature and the elemental concentrations can significantly vary among species, depending on their environment and nutrient availability [33].

A previous investigation analyzed samples of *Ulva lactuca* by energy dispersive X-ray spectroscopy (EDX). It showed values very close to those of the present study for the content of carbon, oxygen, and calcium (26.92%, 48.87%, and 2.00%, respectively) [34]. However, the value of sulfur found in *C. muelleri* is 4.76%, a lower value than that reported for *U. lactuca* (9.11%), which suggests this species could have sulfated polysaccharides in small amounts compared to other species.

### 3.5. Macromolecular Characteristics

The *Mw* and [*η*] of the sample were 4.13 kDa and 4.7 mL/g, respectively (Table 2), where [*η*] measures the polymer’s contribution to the viscosity of a solution when its concentration tends to zero. It will depend on the conformation and the *Mw* of the polymer in solution, determining the physicochemical properties specific to the macromolecule [35].

The *PDI* indicates the chain size distribution present in the polymer [36]. For monodispersed polymers, the *PDI* is 1, whereas *PDI* values lower than 1.2 and higher than 2 are generally interpreted as having a narrow and wide dispersity, respectively [37]. The fraction solubilized in NaNO_3_ showed a *PDI* value of 2.1, indicating a high polydispersity of the chains.

The *Rh* of a sample is the radius of a theoretical sphere with the same mass and density calculated for the sample from its molecular weight and intrinsic viscosity. In this case, the *Rh* value of the CMPS was 1.3 nm. While in the case of the radius of gyration (RG) and the constants *k* and α, it is impossible to obtain reliable values due to isotropic diffusion and the small polysaccharide size [38].

The sulfation degree and Z potential of the CMSP are presented in Table 3. The registered values indicate a small sulfate/carbohydrate ratio in the CMSP, which, in turn, resulted in a positive Z potential value in the macromolecule, in the range reported for other polysaccharides [39].

### 3.6. Cytotoxicity of the CMSP

**Figure 4:**
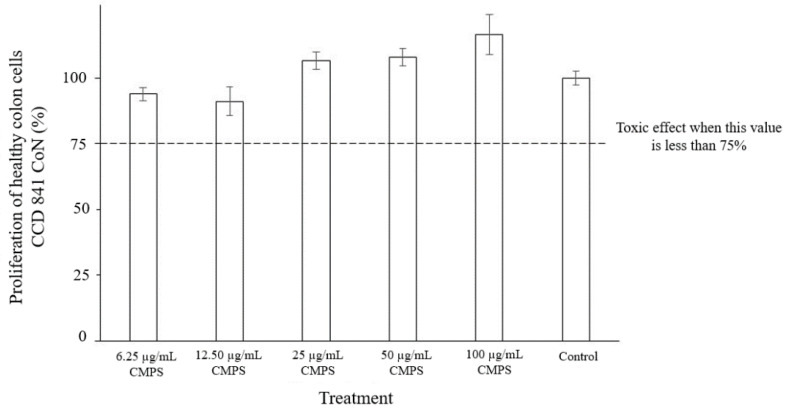
Effect of different concentrations of the CMSP on the proliferation of healthy colon CCD-841 CoN cells in relation to the control treatment.

The CMSP toxicity results are presented in Figure 4. In general, the cell proliferation of the cultures with the CMSP was similar to that of the control. A compound is considered toxic when the cell proliferation value is less than 75% [40]. In the present study, the range of cell proliferation was from 91% to 116% in the cells exposed to the CMSP. These results indicate that the CMSP does not present cytotoxicity on the colon cell line CCD-841 CoN in the range of the evaluated concentrations.

### 3.7. Antioxidant Activity of the CMSP

**Figure 5:**
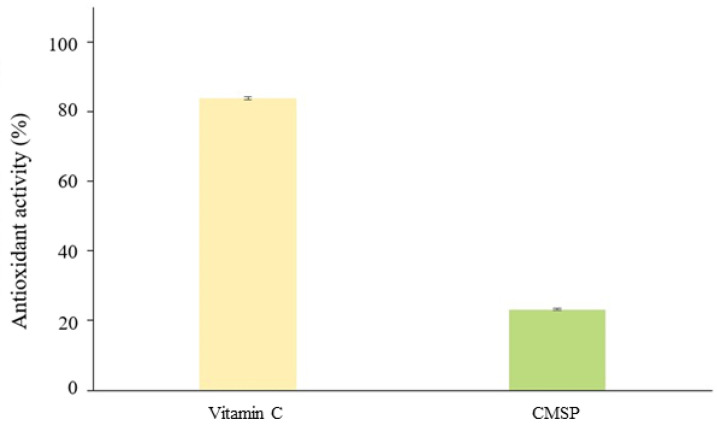
Antioxidant activity of the CMSP in relation to the standard, vitamin C, measured at 20 mg/mL.

The CMSP presented an antioxidant activity of 23%, representing approximately a quarter of the value recorded for vitamin C in the present study (84%) (Figure 5). The percentage of antioxidant activity recorded for the CMSPs (23%) is higher than that reported for the polysaccharides (chrysolaminarins) of the diatom *Odontella aurita* at a concentration of 25 mg/mL (approximately 20%) using the same method (DPPH) used in the present investigation [41].

### 3.8. Glycemic Index (GI)

**Figure 6:**
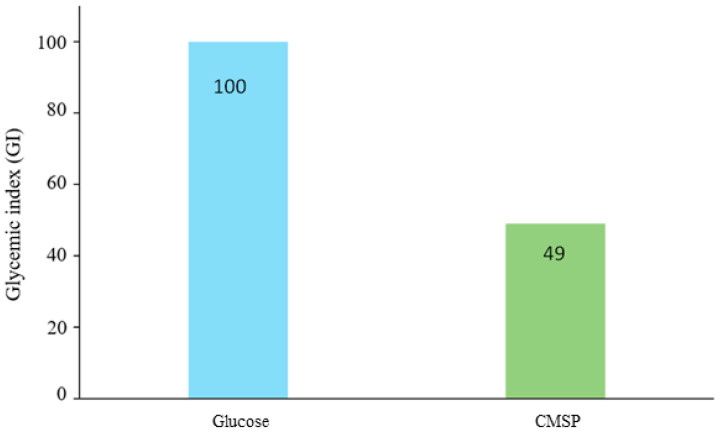
Glycemic index of the CMSP and glucose in Wistar rats. It is calculated from the blood glucose levels in Wistar rats after administration by oral gavage of glucose solution and sulfated polysaccharides of *C. muelleri* at 25 mg/mL.

The GI of the CMSP in Wistar rats in relation to glucose is shown in Figure 6. A greater increase in blood glucose with glucose solution (So) than with CMSP solution can be observed. This result could be related to a complex structure in the CMSP, which has been previously reported for other algal polysaccharides [42]. Using these results, a CMSP GI value of 49 was calculated, which corresponds to a low GI; it is considered a polysaccharide with low glycemic power. This value is close to that reported for sulfated polysaccharides extracted from *Gracilaria chouae*, with GI values of 36.12 and 17.70 [43].

## 4. Discussion

### 4.1. CMSP Yield and Characteristics

Information on polysaccharides from microalgae, particularly diatoms, is scarce. In order to better comprehend the behavior and potential applications of polysaccharides, it is necessary to carry out characterization tests to determine their composition, structure, and macromolecular properties, including rheological properties and molecular weight, since they strongly predict their functionality [6]. Studying new sources of polysaccharides in microalgae offers multiple advantages over working with macroalgae by obtaining polysaccharides under controlled conditions and generating information about new sources and potential applications.

In the present study, the biomass and CMSP yields (2.8 g/L and 2.2% w/w, respectively) were in the range reported for other diatom species, such as *Gomphonema olivaceum* [22] and *Navicula* sp. [23]. Additionally, the composition of the CMSP (Table 1) coincides with that reported for other diatoms, such as *Phaeodactylum tricornutum* [5], which suggests an association of the cell wall proteins and minerals with the polysaccharides. The microstructural characteristics of the CMSPs are similar to those previously described for sulfated polysaccharides from *Navicula* sp. [23].

The CMSPs presented a low degree of sulfation and a positive Z potential value (Table 3). The small sulfate/carbohydrate proportion in the CMSPs may explain why the Z potential does not have a negative charge. A positive Z potential in polysaccharides is considered an advantage from a pharmaceutical point of view when a polysaccharide–cell contact is desired since it favors an interaction with the cell membranes where an opposite charge predominates [39].

### 4.2. Cytotoxicity, Antioxidant Activity, and Glycemic Index of the CMSP

The cytotoxicity of a compound can be evaluated by chemical, biological in vitro tests (cell lines), and bioassays in animals and humans after ingestion of that compound. In vitro studies with cell cultures are helpful diagnostic tools since they can reproduce various physiological states at the laboratory level, facilitating handling and testing conditions [18]. Thus, in the present study, the in vitro cytotoxicity of the CMSP was evaluated to subsequently study its possible potential properties and revalue this microalga and its polysaccharides. In the present study, the colon cell line CCD-841 CoN cells exposed to CMSP registered proliferation values from 91% to 116% (Figure 4), indicating that this polysaccharide does not present cytotoxicity in the range of concentrations evaluated [40].

Antioxidants can help reverse the damaging effects of some free radicals in cells. People with diets rich in antioxidants have a lower risk of cancer, heart disease, and some minor neurological diseases. These effects suggest that compounds with antioxidant activity can prevent some conditions related to poor nutrition and neurodegeneration caused by oxidative stress [44]. Antioxidants have also been associated with preventing premature aging and chronic-degenerative diseases, such as hypertension, rheumatoid arthritis, and the hardening of the arteries, among other conditions. Adequate levels of antioxidants in the diet are essential for good health, which is why there is great interest in these compounds and a constant search for new sources or molecular structures that have this property [45].

The antioxidant activity in the CMSPs was 23% (Figure 5), which is higher than the value reported for polysaccharides from other diatoms, such as *Odontella aurita* [41]. Seaweed polysaccharides have antioxidant activity. Several authors suggest that the structural characteristics of the polysaccharide, such as chain conformation and molecular weight, determine the ability of the molecule to stabilize free radicals [45,46]. The CMSP antioxidant activity may be associated with its low molecular weight (4.13 kDa) and the presence of sulfate in the molecule. As for carrageenan, it has been reported that a lower molecular weight molecule has a higher antioxidant activity than a high molecular weight molecule [46]. This effect may be related to the reducing ends present in the polysaccharide [47].

The glycemic response to carbohydrate-containing foods and their effect on blood glucose can be determined by the glycemic index, which provides a numerical value that represents the effect of a particular food on blood glucose levels. It also provides a quantitative comparison between foods, where most studies use glucose or white bread as the reference, assigning it a score of 100 [48]. Insoluble dietary fiber has been demonstrated to help maintain regular weight and increase intestinal activity, amongst other bioactivities that benefit human health, becoming a part of the functional food family [49]. Therefore, several recent authors have studied the digestion, fermentation, and even the anti-obesity potential of polysaccharides from algae sources [42,43]. The CMSPs registered a GI value of 49 (low GI), which is in the range reported for sulfated polysaccharides from *Gracilaria chouae* (GI values between 36.12 and 17.70) [43]. In this sense, it has been reported that polysaccharides from algae with a low Mw value (4–20 kDa) have a lower GI because they promote insulin secretion, thus, protecting the pancreas and decreasing the risk of insulin resistance development [50]. Additionally, a gradual release and more stable blood glucose levels were observed, which could be related to the complex fiber-like structures of the polysaccharide.

## 5. Conclusions

The present study confirmed that the sulfated polysaccharides from the microalga *Chaetoceros muelleri* (CMSPs) do not exert cytotoxicity on healthy human colon cells. Additionally, they present antioxidant activity and a low glycemic index. Although the results of the antioxidant activity are encouraging, they did not exceed the Vitamin C value. However, it should be noted that the CMSP concentrations used in the present study for both the antioxidant activity and the glycemic index were similar, which could add value to these polysaccharides by keeping low glycemic levels and exerting antioxidant activity at the same time. Nevertheless, further studies should be considered to confirm this premise. In general, the results indicate that the macromolecular characteristics, especially the low Mw, enhance the bioactive properties of CMSPs. In this regard, CMSPs would be an excellent option for biotechnological applications, such as food additives or neutraceuticals, particularly those related to human health. These initial results on CMSPs can start further investigations into more advanced characterizations, such as monomeric composition, to better understand chain conformation and functionality. In addition, implementing diatom cultures on a large scale under controlled conditions for optimizing functional polysaccharide production might prove an important area for future research.

## Figures and Tables

**Table 1:** Composition of the polysaccharides extracted from *C. muelleri*.

Component	% (w/w)
Total carbohydrates	78 ± 0.3
Protein	5 ± 1.0
Ash	17 ± 0.2

Mean value ± SD of the triplicate determinations.

**Table 2:** Macromolecular characteristics of the CMSP from *C. muelleri*.

*Mw* (kDa)	4.13
*Mn* (kDa)	1.94
*PDI* (*Mw/Mn*)	2.12
[*η*] (mL/g)	4.68
*Rh* (nm)	1.33

*Mw*, weight-average molar mass; *Mn*, number-average molar mass; *PDI*, polydispersity index; [*η*], intrinsic viscosity; *Rh*, hydrodynamic radius.

**Table 3:** Degree of sulfation and Z potential of the CMSP.

Characteristic	Value
Sulfation degree (sulfate/carbohydrate ratio)	0.10 ± 0.01
Z Potential (mV)	+1.91 ± 0.3

Mean value ± SD of the triplicate determinations.

## Data Availability

Not applicable.
